# Exosomal miR-130b-3p targets SIK1 to inhibit medulloblastoma tumorigenesis

**DOI:** 10.1038/s41419-020-2621-y

**Published:** 2020-06-01

**Authors:** Saihua Huang, Ping Xue, Xiao Han, Caiyan Zhang, Lan Yang, Lijuan Liu, Xiang Wang, Hao Li, Jinrong Fu, Yufeng Zhou

**Affiliations:** 1 Institute of Pediatrics, Children’s Hospital of Fudan University, and the Shanghai Key Laboratory of Medical Epigenetics, International Co-laboratory of Medical Epigenetics and Metabolism, Ministry of Science and Technology, Institutes of Biomedical Sciences, Fudan University, 200032 Shanghai, China; 2 Department of Neurosurgery, Children’s Hospital of Fudan University, Shanghai, China; 3 NHC Key Laboratory of Neonatal Diseases, Fudan University, 201102 Shanghai, China

**Keywords:** CNS cancer, Diagnostic markers

## Abstract

Exosomes are an important carrier for cell communication. miRNAs in exosomes are potential biomarkers and therapeutic targets in different types of cancer. However, the role of exosomal miRNAs in medulloblastoma (MB) patients is largely unknown. In this study, we reported that there was a higher level of miR-130b-3p in exosomes derived from MB patient plasma compared with exosomes from healthy control plasma. Exosomes from MB patient plasma could transfer miR-130b-3p to an MB cell line and played suppressor roles for cell proliferation. miR-130b-3p suppressed MB tumorigenesis by targeting a previously unknown target, serine/threonine-protein kinase 1 (SIK1), through the p53 signaling pathways. In addition, we found an unreported role of SIK1 in promoting MB tumor growth and an SIK1 inhibitor could inhibit MB cell proliferation. This research provides new insight into the molecular mechanism of MB and may provide a new therapeutic strategy for MB treatment.

## Introduction

Medulloblastoma (MB) is the most common malignant brain tumor in children, accounting for 20−30% of children with central nervous system malignancies^1^, and the peak incidence is between 3 and 4 years and 8 and 9 years of age^2^. The present clinical treatments for MB involve a combination of surgery, cranio-spinal radiotherapy (in children ≥3 years of age) and chemotherapy, but these treatments are often associated with significant side effects^3–5^. Major challenges for patients and physicians include a lack of early diagnostic biomarkers, the strong tendency of this cancer to metastasize along the cerebrospinal axis, and a lack of precision treatment options based on the tumor molecular background^6,7^. Therefore, early diagnostic biomarkers, a comprehensive understanding of the molecular mechanisms involved in MB tumorigenesis, and methods to regulate these are urgently needed.

Exosomes are secreted by all living cells, and are small membrane vesicles of 50−150 nm in diameter containing microRNAs (miRNAs), protein, lipids, DNA and mRNA^8,9^. Exosomes are an important carrier in cell communication^10^. miRNAs are highly conserved, single-stranded RNA molecules of 19−24 nucleotides long^11^. They regulate genes by binding to the 3′ untranslated region (3′UTR) of target messenger RNA^12,13^. Exosomal miRNAs are emerging as novel regulators of cellular function^14–16^. Exosomal miRNAs moving from cancer cells to endothelial cells could promote tumor metastasis^17^. Exosomal miRNAs secreted from immune cells were shown to exert an antiproliferative effect on tumor cells^14^. Therefore, miRNAs in exosomes are potential biomarkers and therapeutic targets in different types of cancer.

It has been reported that the miR-130 family plays an important role in the development of cancer, but the results vary in different cancers^18–20^. Yeung et al.^21^ showed that miR-93/miR-130b could promote tumor growth by inhibiting *Tp53INP1* expression. However, it has been found that miR-130b-3p could directly target the Notch ligand Delta-like 1 (*DLL1*) and inhibit breast carcinoma cell invasion and migration^22^. It has been suggested that the upregulation of miR-130b-3p contributed to the development of thyroid adenomas by targeting the *CCDC6* gene^23^. In a pilot RNA-seq analysis of exosome in the plasma of MB patients (GSE123376), we found exosomal miR-130b-3p was elevated in the plasma of MB patients. However, the effects of miR-130b-3p derived from exosomes in MB remain unknown. In the current study, we confirmed that there was a higher level of miR-130b-3p in exosomes derived from the MB patient plasma than in those from healthy control plasma. We investigated the tumor suppressor role of miR-130b-3p in MB in vitro and in vivo by targeting a previously unknown target, serine/threonine-protein kinase 1 (SIK1), through the p53 pathways. Our research provides new insights into the molecular mechanism of MB and may offer new therapeutic strategies for MB treatment.

## Materials and methods

### Patients and samples

Blood samples from MB patients and healthy donors were obtained from Children’s Hospital of Fudan University. The study protocol was approved by the hospital institutional review board and written informed consent was obtained from each participant. Blood plasma was retrieved from whole-blood samples (4 mL) via centrifugation at 400 × *g* for 10 min at 4 °C and aliquoted and stored at −80 °C until analysis. The relevant characteristics of patients are summarized in Supplementary Table 1.

### Cell lines and cell culture

The human MB cell line, Daoy, was purchased from the Shanghai Institute of Cell Biology, Shanghai, China. The cells were cultured in MEM (Minimum Essential Medium) supplemented with 10% fetal bovine serum (FBS), 1% sodium pyruvate, nonessential amino acids and penicillin-streptomycin. The D283 Med cells were cultured in RPMI 1640 media. Cells were maintained at 37 °C in a humidified atmosphere containing 5% CO_2_.

THP-1 monocytes were maintained in RPMI 1640 media supplemented with 10% heat-activated FBS, penicillin (100 U/mL) and streptomycin (100 μM). HMO6 cells, an immortalized human microglial cell line, were cultured in DMEM (Dulbecco’s Modified Eagle Medium) high-glucose medium. For exosome collection, THP-1 cells (1 × 10^7^) were plated in 10 cm dishes with complete culture media overnight, and transfected with miR-130b-3p mimic or NC. At 48 h, washed twice with PBS, and media was replaced with FBS free media and incubated overnight. Media was collected at 24 h for exosome purification. For exosome purification, HMO6 cells were cultured as mentioned above for THP-1 cells.

### Exosome isolation, identification and labeling

Exosomes were isolated from the plasma of healthy control subjects, MB patients and culture supernatant of THP-1 or HMO6 cells with ultracentrifugation method by series of centrifugation at 4 °C: 300 × *g* for 10 min, 2000 × *g* for 10 min to remove cellular debris and large apoptotic bodies, 100,000 × *g* for 70 min to precipitate exosomes, and 100,000 × *g* for 70 min to obtain purer exosomes. Exosomes were quantified by a BCA Protein Assay Kit (Takara).

For transmission electron microscopy (TEM) analysis, isolated exosomes were fixed with 4% paraformaldehyde and 4% glutaraldehyde in 0.1 M phosphate buffer (pH 7.4) and then placed on a carbon-coated copper grid and immersed in a 2% phosphotungstic acid solution for examination (JEM-1200EX; JEOL Ltd., Japan). Western blot analysis was used to detect biomarkers of exosomes including CD9 and CD63, and the Golgi marker GM130 was used as a negative control. To observe the cellular uptake of exosomes, purified exosomes were labeled using a PKH67 labeling kit (Sigma-Aldrich). After co-culture with labeled exosomes for 12 h, Daoy cells were fixed and stained using Hoechst33258. Images were obtained using a Lecia TCS-SP5 LSM.

### Treatment of Daoy and D283 with exosomes

The Daoy and D283 cells were seeded into 24-well plates or 96-well plates overnight, and then 50 μg/mL of exosomes secreted from the plasma of healthy control subjects or MB patients, THP-1-transfected miR-130b-3p mimic or NC was added into per well. After being incubated for 24 h at 37 °C, the cells were harvested for cell survival assays and RT-qPCR.

### Transfection

The miR-130b-3p mimic, corresponding negative control, si-SIK1 and nonspecific siRNA negative control used herein were purchased from GenePharma (Shanghai, China). The transfection was conducted using Lipofectamine RNAiMAX (Invitrogen, Carlsbad, CA, USA) at a final concentration of 200 nM in accordance with the manufacturer’s instructions. The sequences of mimic and siRNA are listed in Supplementary Table 2.

### RNA isolation and real-time quantitative PCR

Total RNA was extracted from the cells or tissues using TRIzol(Invitrogen, USA) in accordance with the manufacturer’s protocol. For the expression level of exosomal miRNAs verification, commercial kit was used to extract total RNA from exosomes (exoRNeasy Serum/Plasma Maxi Kit (50), Qiagen). The expression of miRNAs was determined using TaqMan microRNA assay kits (ABI, USA) with U6 as an internal control. To analyze the mRNA levels of SIK1, total RNA was reverse transcribed with oligo dT primers using the PrimeScript RT Reagent kit (Takara, Dalian, China). Actin served as an internal control. Relative RNA expression levels were quantified using the 2^−ΔΔCT^ method. The sequences for primers are listed in Supplementary Table 3.

### Cell growth assay

To assay cell proliferation, the cell counting kit-8 (CCK-8) colorimetric assay (DOJINDO Molecular Technologies, Inc., Kumamoto, Japan) was performed. Daoy cells were placed in 96-well plates and cultured in the complete medium. After incubation, the cells were transfected with 200 nM of the miR-130b-3p mimic/miR-NC or si-SIK1/si-NC. D283 Med cells were transfected with the lentiviral vector for delivery of miR-130b-3p and the corresponding negative control purchased from GenePharma (Shanghai, China). Daoy cells were treated using an inhibitor of SIK1 (HG-9-91-01) for 1 h, and then tested using CCK-8 kit. Each sample was assayed in triplicate. Cell viability was determined at 0, 24, 48 and 72 h using the CCK-8 assay. The optical density of each well was assessed using a Microplate reader at 450 nm to determine cell viability. Each experiment was performed in triplicate.

### Colony-formation assay

We also performed a colony-forming ability assay to assess Daoy cell proliferation. Followed by transfection with the miR-130b-3p mimic, si-SIK1, or NC, Daoy cells were seeded into 60 mm culture dishes at a density of 500 cells/dish and cultured in a 5% CO_2_ incubator at 37 °C. After 2 weeks, the cells were stained using crystal violet.

### Cell apoptosis assays

Daoy cells were cultured in six-well plates for 48 h after miRNA and siRNA transfection and D283 Med cells were transfected with the lentiviral vector for delivery of miR-130b-3p and the corresponding negative control, and then treated using 100 μmol H_2_O_2_ overnight. The apoptosis assays were evaluated through flow cytometry using the FITC Annexin V Apoptosis Detection Kit I (BD, San Diego, CA, USA) in accordance with the manufacturer’s instructions.

### Wound-healing assays

For wound-healing assays, transfected MB cells were grown to 100% confluence in six-well plates, after which scratch-wounds were created in the monolayers using a sterile 200 μL pipette tip. Photographs were taken using phase-contrast microscopy (Leica, Germany) immediately and 24 h after wound creation.

### Cell invasion assay

Cell invasion assay was performed using Matrigel-coated transwell chambers (8 μm pore size; Costar). A total of 100 μL transfected cell suspension (2 × 10^4^ cells/mL) was added to the upper chamber of the transwell plates. A total of 500 μL complete medium was added to the bottom chambers. After incubation for 24 h at 37 °C, nonmigrating cells were removed using cotton swabs. The cells that permeated the Matrigel-coated membrane and migrated to the bottom were fixed using paraformaldehyde and then stained using crystal violet.

### Luciferase reporter assay

The SIK1 3′UTR, including wild-type miR-130b-3p binding sites, and the mutated SIK1 3′UTR were respectively cloned into the downstream of the firefly luciferase gene within the psiCHECK-2 vector (Promega). For reporter assays, HEK-293T cells were seeded in 96-well plates in triplicate and allowed to settle for 24 h. The cells were then cotransfected with 10 ng firefly luciferase reporter plasmid and an equal amount (200 nM) of miR-130b-3p mimic or scrambled negative control RNA using Lipofectamine 2000 (Invitrogen, Carlsbad, CA, USA). At 24 h post transfection, the cells were assayed using a luciferase assay kit (Promega, Madison, WI, USA).

### Western blotting assay

Cells were washed in PBS and lysed using RIPA lysis buffer supplemented with a protease phosphatase inhibitor. Total protein was quantified using a BCA Protein Assay Kit (Takara), and equal amounts of whole-cell lysates were resolved through SDS-polyacrylamide gel electrophoresis and transferred to polyvinylidene difluoride membranes (Bio-Rad, USA). The blots were blocked in bovine serum albumin (5% w/v in PBS + 0.1% Tween20) for 1 h at room temperature and immune stained using antibodies at 4 °C overnight. The membranes were incubated using primary antibodies and horseradish peroxidase-conjugated secondary antibodies. Enhanced chemiluminescence assays were used to detect the signal. The antibodies against CD9 (1:1000), GM130 (1:1000), p53 (1:1000), Bcl-2 (1:1000) were purchased from Cell Signaling Technology, Inc. Anti-CD63 (1:1000) and anti-SIK1 (1:1000) were purchased from Abcam.

### Vector constructs and lentiviral production

The lentiviral vectors for delivery of miR-130b-3p and the corresponding negative control were purchased from GenePharma (Shanghai, China). sh-SIK1 was amplified and cloned into the pLenti vector after double digestion by *Bam*HI and *Eco*RI to form sh-SIK1 pLenti vector and then the constructed vector was sequenced (Sangon Biotech, Shanghai, China). For stable transfection, cells were transfected with sh-SIK1 or negative control (sh-GFP) lentiviral particles, in accordance with the manufacturer’s protocol. The Daoy cells were transfected in growth media containing 1 µg/mL puromycin and until the green fluorescent protein included in the plasmid vector was visualized. Total RNA in stably transfected cells was extracted to detect the efficiency of stable transfection.

### Animal experiments

Male BALB/c nude mice (6 weeks old, obtained from the SLRC Laboratory Animal Center, Shanghai, China) were subcutaneously injected with 5 × 10^6^ Daoy-miR-130b-3p cells, Daoy control cells, sh-GFP cells and sh-SIK1 cells. The injected cells were suspended in 100 μL of PBS/matrigel (v/v, 1:1). Tumor volumes were measured using calipers and calculated using the following formula: volume = length × (width)^2^/2. The curve of tumor growth was drawn based on tumor volume and corresponding time (days) after treatment. When the animals were terminated, the tumor tissues were removed and weighed. The animal assay complied with the Guide for the Care and Use of Laboratory Animals and was approved by the Animal Studies Committee of the Children’s Hospital of Fudan University.

### Immunohistochemistry, hematoxylin and eosin staining and fluorescence in situ hybridization

Dissected tissues were formalin-fixed, paraffin-embedded, sectioned and stained using hematoxylin and eosin in accordance with standard histopathological techniques. For immunohistochemistry (IHC), sections were incubated with primary antibodies against Ki67 and SIK1, and secondary antibodies, followed by staining and imaging.

The work flow of fluorescence in situ hybridization (FISH) (GenePharma, Shanghai, China) for miR-130b-3p is described below. In brief, paraffin sections were deparaffinized by xylene and gradient alcohol (100%, 95%, 90%, 80%, 70%). The slices were treated by Proteinase K and denaturation solution. Hybridization: (1) Probe dilution: Add DEPC water to the probe dry powder product to obtain a stock solution with a concentration of approximately 100 μM. (2) Preparation of probe mixed solution. (3) Prepare a wet box, place the slices horizontally, add 100 μL of the denatured probe mixture to each slice and incubate at 37 °C for 12−16 h. Stringency wash: This step is essential for removing the overload probes and nonspecific probe binding. The slices were incubated by DAPI for nuclear staining.

### Statistical analysis

All data are shown as the means ± SEM. Student’s *t* test was used for comparisons between two groups, and one-way analysis of variance was used for multiple comparisons. *p* < 0.05 was considered to indicate statistical significance (*p < 0.05, **p < 0.01, ***p < 0.001).

## Results

### miR-130b-3p is upregulated in exosomes derived from MB patient plasma and can be transferred to tumor cells through exosomes

**Fig. 1:**
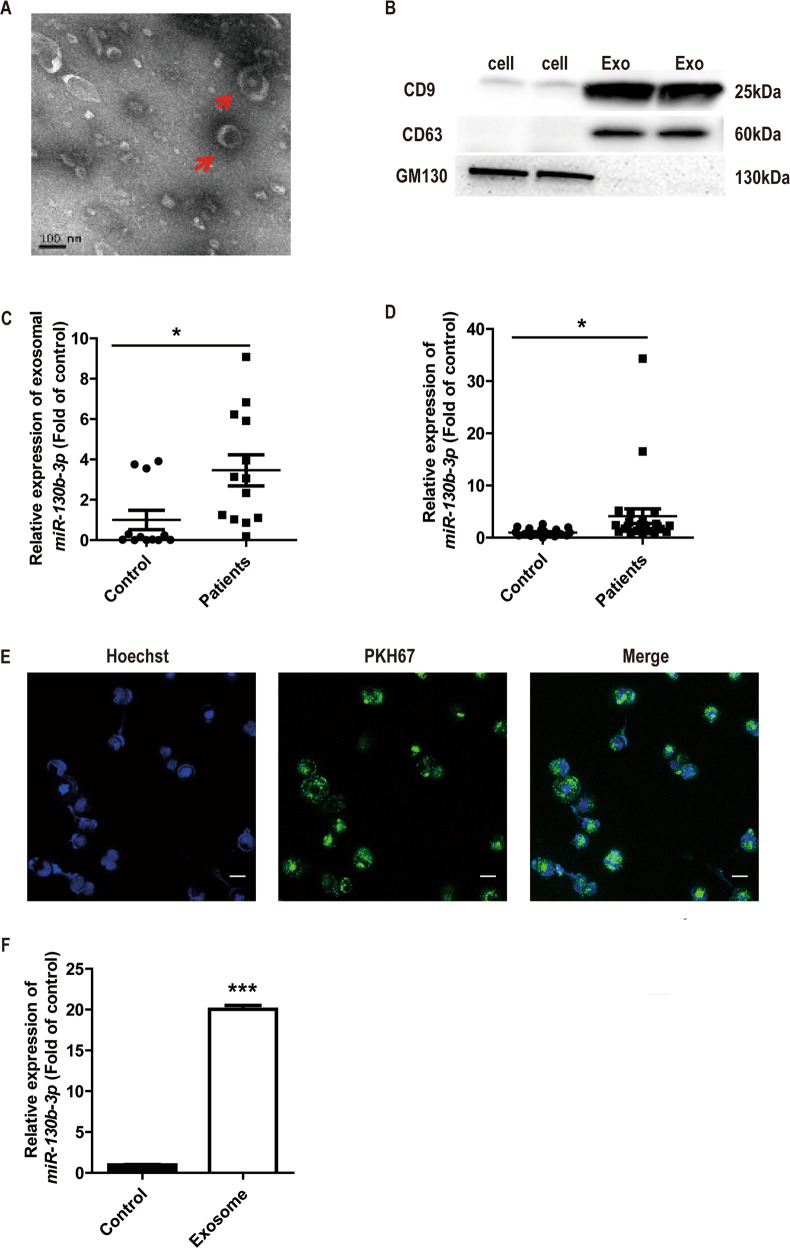
miR-130b-3p can be transferred to tumor cells through exosomes. **a** The exosomes was assessed using transmission electron microscopy. Scale bar, 100 nm. **b** Western blot analysis of the exosomal marker, CD9 and CD63; Golgi marker, GM130. **c** Exosomal miR-130b-3p expression in healthy and MB patient plasma detected by RT-qPCR. **d** The expression of miR-130b-3p in healthy and MB patient PBMCs detected by RT-qPCR. **e** Exosomes were isolated from MB patient plasma, dyed with PKH67 (green) and cocultured with Daoy cells for 12 h, then dyed with Hoechst33258 (blue) and viewed with confocal microscopy (original magnifications ×200). **f** The expression of miR-130b-3p in Daoy cells cocultured with the exosomes isolated from MB patient plasma, detected by RT-qPCR. Data represent means ± SEMs from three independent experiments. *p < 0.05, ***p < 0.001.

To identify differentially expressed exosomal miRNA in MB, we previously analyzed the miRNA expression profiles of exosomes derived from MB patient plasma by RNA-seq (GSE123376, submitted in other draft), and found miR-130b-3p was highly expressed in exosomes from MB patient plasma compared with healthy controls (*n* = 3, fold-change >1.5) (Supplementary Fig. S1). Subsequently, we evaluated the expression of exosomal miR-130b-3p derived from MB patients and healthy control plasma in a larger population of samples. Exosomal fractions were prepared from MB patient plasma by ultracentrifugation. TEM revealed that the exosomes showed typically round shapes, ranging in size between 50 and 150 nm (Fig. 1a). To confirm the identity of the isolated exosomes, we examined some specific exosome markers, CD9 and CD63. As shown in Fig. 1b, CD9 and CD63 were highly enriched in the isolated exosomes. Conversely, the negative maker of GM130 (Golgi marker) was almost undetectable in the isolated exosomes, but highly expressed in cells (Fig. 1b). It was found the expression of exosomal miR-130b-3p in MB patients was significantly higher than that of control (Fig. 1c). We also detected the expression of miR-130b-3p in the peripheral blood mononuclear cells (PBMCs) from MB patients and healthy controls by RT-qPCR, and found that the expression of miR-130b-3p in the PBMCs of MB patients was notably higher than that of healthy control (Fig. 1d). We further evaluated the expression of miR-130b-3p in MB tissues and adjacent noncancerous tissues (the clinical characteristics of the patients are shown in Supplementary Table 1), and found that the expression level of miR-130b-3p was significantly lower in tumor tissues than in nontumor tissues (Supplementary Fig. S2a).

It was reported exosomes played very important roles in communication between cells, and miRNAs were reported to be responsible for these roles of exosomes. As mentioned before, miR-130 family plays important roles in tumorigenesis; we thus hypothesized that exosomal miR-130b-3p in plasma could transfer into MB cells. Exosomes labeled with a fluorescent dye, PKH67, were added to Daoy culture for 12 h. After incubation, fluorescence microscope imaging showed the presence of PKH67 spots in recipient Daoy cells, suggesting that labeled exosomes were delivered to Daoy cells (Fig. 1e). We assessed the expression of miR-130b-3p in Daoy cells that were cocultured with exosomes derived from plasma. The expression of miR-130b-3p was higher in cocultured with exosomes isolated from the MB patient plasma (Fig. 1f). Therefore, the data showed that exosomes from MB patient plasma could transfer miR-130b-3p into tumor cells.

### Exosomal miR-130b-3p inhibit MB cell proliferation in vitro

**Fig. 2:**
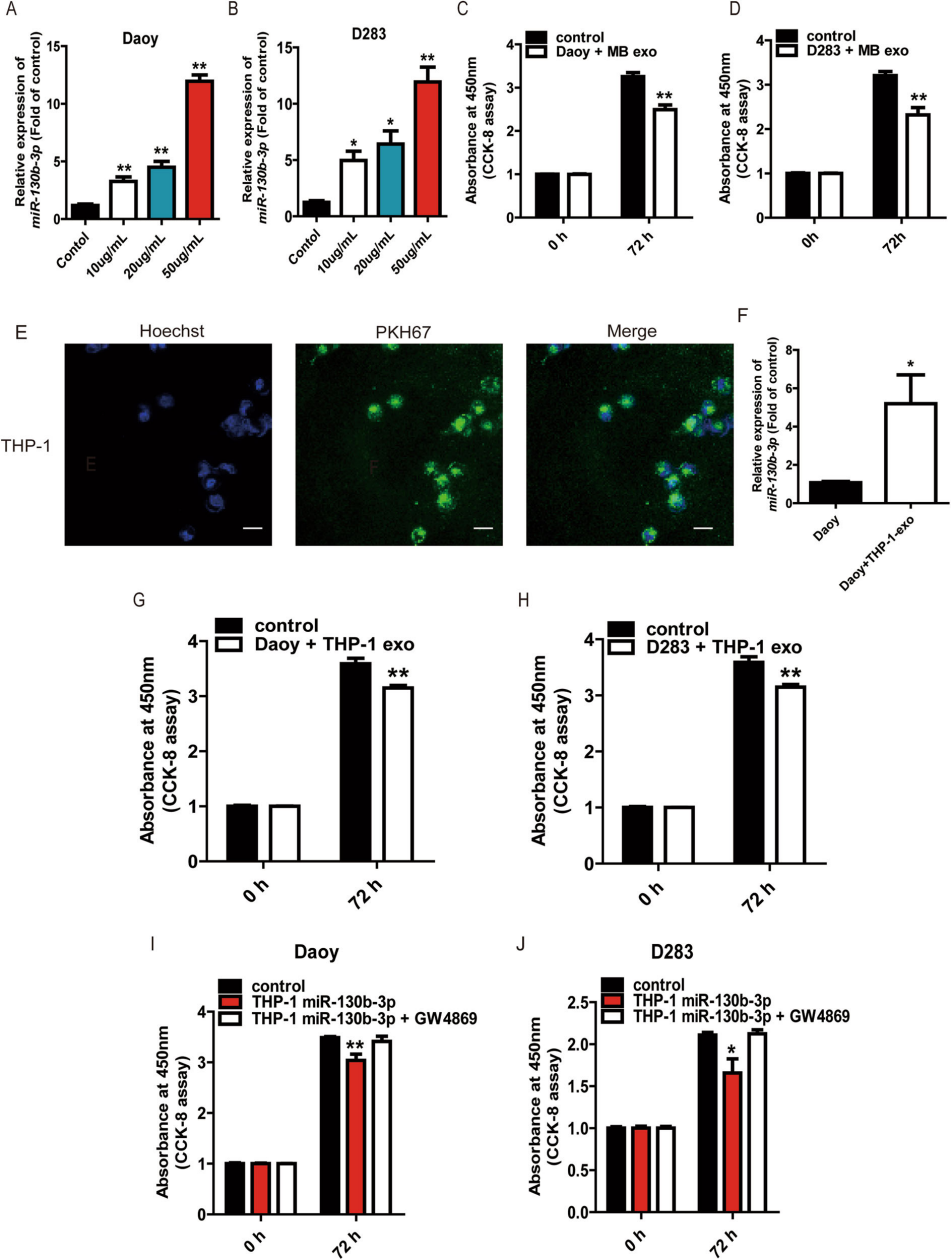
Exosomal miR-130b-3p inhibit MB cell proliferation in vitro. **a**, **b** RT-qPCR assay was used to detect the expression of miR-130b-3p in Daoy and D283 cocultured with the different concentrations of exosomes (10, 20, 50 μg/mL) collected from MB patient plasma. **c**, **d** The proliferative ability of Daoy and D283 treated with the different concentrations of exosomes derived from MB patient plasma by CCK-8. **e** Exosomes were isolated from culture supernatant from THP-1, dyed with PKH67 (green) and cocultured with Daoy, then dyed with Hoechst33258 (blue) and viewed with confocal microscopy (original magnifications ×200). **f** The expression of miR-130b-3p in Daoy cocultured with the exosomes collected from THP-1 culture supernatant, detected by RT-qPCR. The proliferative ability of Daoy (**g**) and D283 (**h**) treated with the exosomes derived from supernatant of THP-1 transfected with miR-130b-3p mimic or NC was tested by CCK-8. Cell proliferation of Daoy (**i**) and D283 (**j**) treated with supernatant of THP-1 transfected with miR-130b-3p mimic or NC and GW4869 was assessed by CCK-8. Data are presented as mean ± SEM. *p < 0.05, **p < 0.01.

To identify the effect of exosomal miR-130b-3p on MB proliferation, we first extracted exosomes from the MB patient plasma and healthy control, which were used to treat the Daoy and D283. We tested the expression of miR-130b-3p in Daoy and D283 that were cocultured with different concentrations of exosomes derived from MB patient plasma by RT-qPCR. The expression of miR-130b-3p was the highest in these cells which were cocultured with 50 μg/mL exosomes (Fig. 2a, b). It was found that there was a significantly lower proliferation rate when Daoy and D283 incubated with exosomes derived from MB patient plasma than with control (Fig. 2c, d).

In our study, we found miR-130b-3p was upregulated in the plasma exosome and PBMCs while it was downregulated in tumor sites (Fig. 1c, d; Supplementary Fig. S2a). A possible reason for this phenomenon was that exosomal miR-130b-3p in the plasma might be derived from monocytes or macrophages rather than tumor cells. Exosomes derived from macrophage account for a large proportion of circulating microvesicles in blood^24^. As THP-1 (a human leukemia monocytic cell line) could be readily differentiated into macrophages, and released high levels of exosomes, we used the THP-1 monocyte-derived macrophages as a model cell line for generation of immune cell exosomes^25^. We extracted the exosomes from THP-1-transfected with miR-130b-3p mimic or NC, then cocultured with Daoy and found that exosomes from THP-1 cells could also transfer into MB cells, and the expression of miR-130b-3p was higher in cocultured with exosomes derived from THP-1 (Fig. 2e, f). After coculturing of Daoy or D283 with the exosomes of THP-1, we found that the cell proliferation was inhibited by exosomes from miR-130b-3p-overexpressed THP-1 cells (Fig. 2g, h). To further confirm that this phenomenon was caused by exosomes, we used the neutral sphingomyelinase inhibitor GW4869 to treat THP-1 overexpressing miR-130b-3p to inhibit exosome secretion. The tumor cell proliferation was inhibited when the cells were cocultured with the miR-130b-3p-overexpressed THP-1 medium, while there was no change in the GW4869-treated medium (Fig. 2i, j). These data indicated that the effects on cell proliferation were at least partly due to the presence of exosomes, and that these exosomes from THP-1 could be eliminated by GW4869 treatment. Same as THP-1, we found HMO6 (a type of glial cell, equivalent to a macrophage in the brain) could secret exosomes containing miR-130b-3p (Supplementary Fig. S3a, b). We also found there was higher number of CD68 + cells in the MB tissues (Supplementary Fig. S3c). Together, these data showed that miR-130b-3p could be passed by exosomes, and exosomal miR-130b-3p reduced cell growth.

### miR-130b-3p functions as a tumor suppressor for MB cells in vitro

**Fig. 3:**
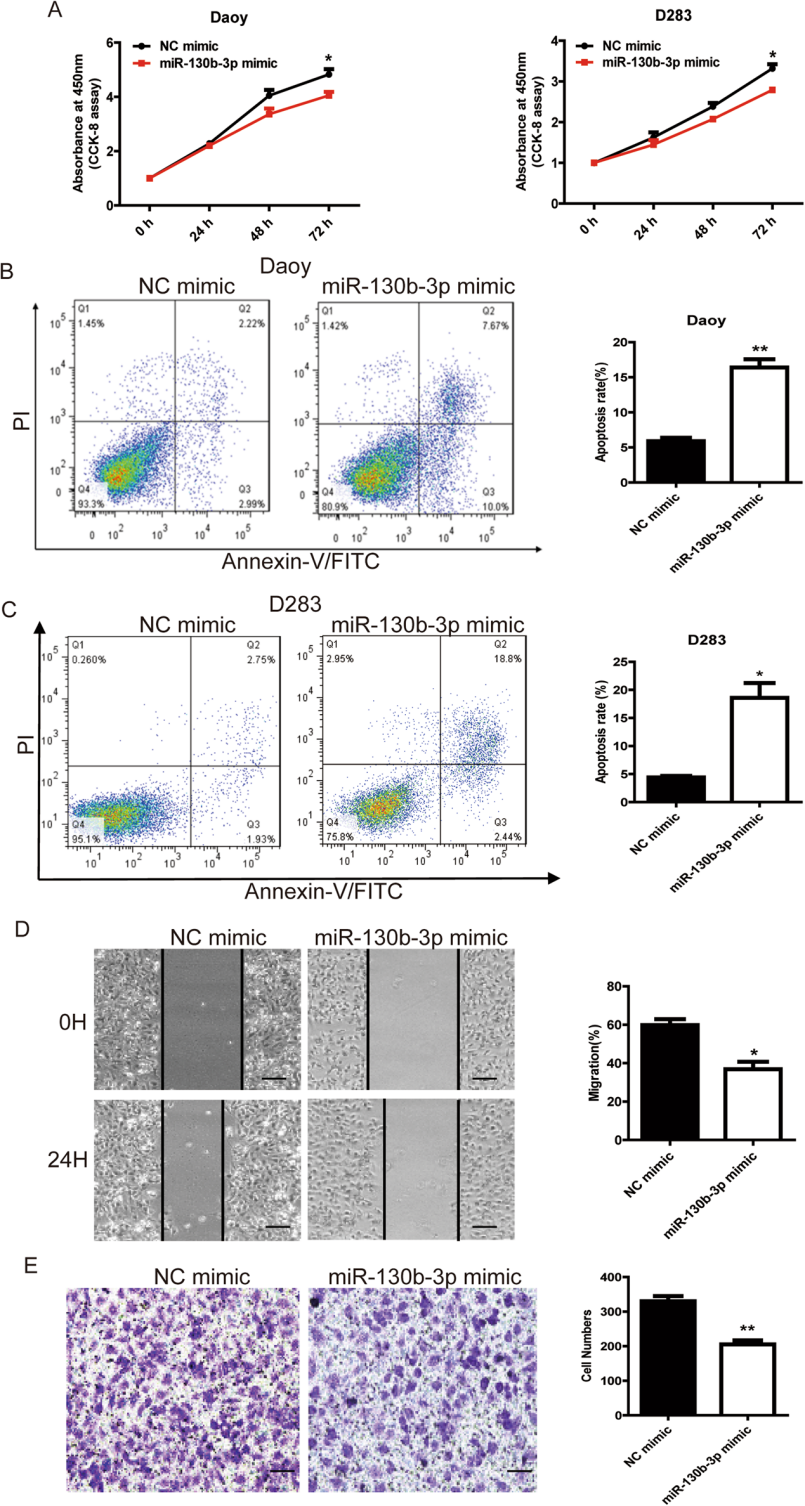
miR-130b-3p functions as a tumor suppressor of MB cells in vitro. **a** The proliferative ability of MB cells transfected with miR-130b-3p mimic or NC was tested through CCK-8. **b**, **c** Cell apoptosis was detected through flow cytometry in MB cells transfected with miR-130b-3p mimic or negative control. **d** Cell migration was detected through a wound-healing assay in MB cells transfected with miR-130b-3p or NC (original magnifications ×200). **e** Cell invasion analysis was performed through transwell invasion assays in MB cells transfected with miR-130b-3p or NC (original magnifications ×200). Data represent means ± SEMs from three independent experiments. *p < 0.05, **p < 0.01.

To investigate the potential biological functions of intracellular miR-130b-3p in MB, we performed gain-of-function analysis using miR-130b-3p mimic to assess its role in cell proliferation and apoptosis. The overexpression of miR-130b-3p in Daoy and D283 Med cells, achieved through transfection with miR-130b-3p mimic, inhibited cell growth (Fig. 3a) and colony formation (Supplementary Fig. S4a). In addition, miR-130b-3p mimic markedly increased the rate of apoptosis in Daoy and D283 Med cells compared with the negative control (Fig. 3b, c). We also examined the effects of miR-130b-3p on cell migration ability by performing a wound-healing assay. As D283 Med cells are suspension cells, we did not detect the motility. Daoy cells with miR-130b-3p overexpression showed a decreased ability to close the wound compared with cells transfected with negative control mimic, suggesting that miR-130b-3p inhibited cell migration (Fig. 3d). In addition, the effect of miR-130b-3p on cell invasion, a hallmark of malignancy, was examined using a transwell assay. miR-130b-3p significantly reduced cell invasion of Daoy cells by 52% (Fig. 3e). Therefore, miR-130b-3p considerably suppressed the migration and invasiveness of MB cells.

### SIK1 is a target of miR-130b-3p

**Fig. 4:**
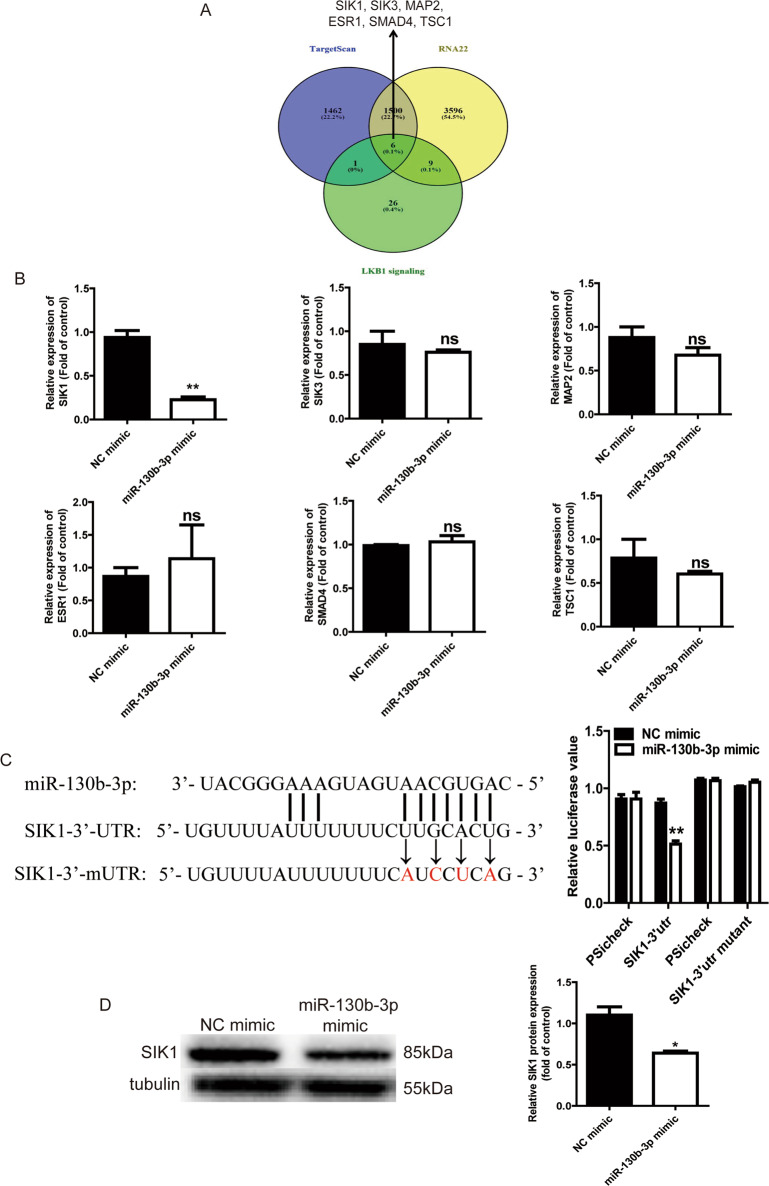
SIK1 is a target of miR-130b-3p. **a** Venn diagrams showing the number of potential miR-130b-3p targets. **b** RT-qPCR analysis of six potential miR-130b-3p target genes in MB cells with miR-130b-3p overexpressed. **c** The potential binding sites of miR-130b-3p within the SIK1 mRNA 3′-UTR and 3′-mUTR. Luciferase reporter gene assays were used to analyze the effect of miR-130b-3p on luciferase activity. **d** SIK1 protein levels were detected through western blotting in MB cells transfected with miR-130b-3p mimic. NC negative control. Data represent means ± SEMs from three independent experiments. *p < 0.05, **p < 0.01.

MicroRNAs often target the 3′UTRs of mRNAs to regulate their stability and translation efficiency. Using two different target predicting algorithms including Target Scan and RNA22, 1506 mRNAs were predicted to be the target of miR-130b-3p (Fig. 4a). LKB1 (also known as Stk11) is a serine/threonine protein kinase that regulates growth and metabolism through adenosine monophosphate-activated protein kinase (AMPK) and 12 other closely related kinases. Studies have reported that LKB1 signaling plays important roles in cell proliferation and migration. There are 42 genes involved in LKB1 signaling events. We overlapped the Target Scan, RNA22 and LKB1 signaling events to get six candidate genes (*SIK1*, *SIK3*, *ESR1*, *SMAD4*, *MAP2* and *TSC1*) (Fig. 4a). Next, we compared the mRNA levels of these genes in Daoy cells transfected with miR-130b-3p mimic and found that SIK1 was significantly downregulated among these six genes (Fig. 4b). To further investigate whether the SIK1 gene is a direct target of miR-130b-3p in MB cells, a dual luciferase reporter assay was performed using a reporter vector (psicheck2). Luminescence was measured after cotransfection with miRNA mimic and plasmid vectors in HEK293T cells. After cotransfection for 24 h, the negative control miRNA did not decrease the luciferase activity. At the same time, transfection with the miR-130b-3p mimic did not reduce the luciferase activity produced by the negative control vector without the SIK1 3′UTR sequence. However, cells transfected with miR-130b-3p mimic and the SIK1 3′UTR vector showed markedly lower luciferase activity compared with the control and the negative control. In contrast, cotransfection of miR-130b-3p with the SIK1-3′UTR mutant showed no remarkable change in luciferase activity, indicating the direct binding between miR-130b-3p and the SIK1 3′UTR (Fig. 4c). The western blot analyses confirmed that miR-130b-3p also suppressed the endogenous expression of SIK1 at the protein level in MB cells (Fig. 4d). This suggests that SIK1 is a target of miR-130b-3p. Next, we analyzed SIK1 expression levels in 25 MB tumor tissue specimens and 15 nontumor tissues through RT-qPCR, and the results showed that SIK1 mRNA expression levels were remarkably upregulated in the MB tissue specimens compared with the nontumor tissues (Supplementary Fig. S2b).

### miR-130b-3p suppresses MB cell tumorigenesis through SIK1

**Fig. 5:**
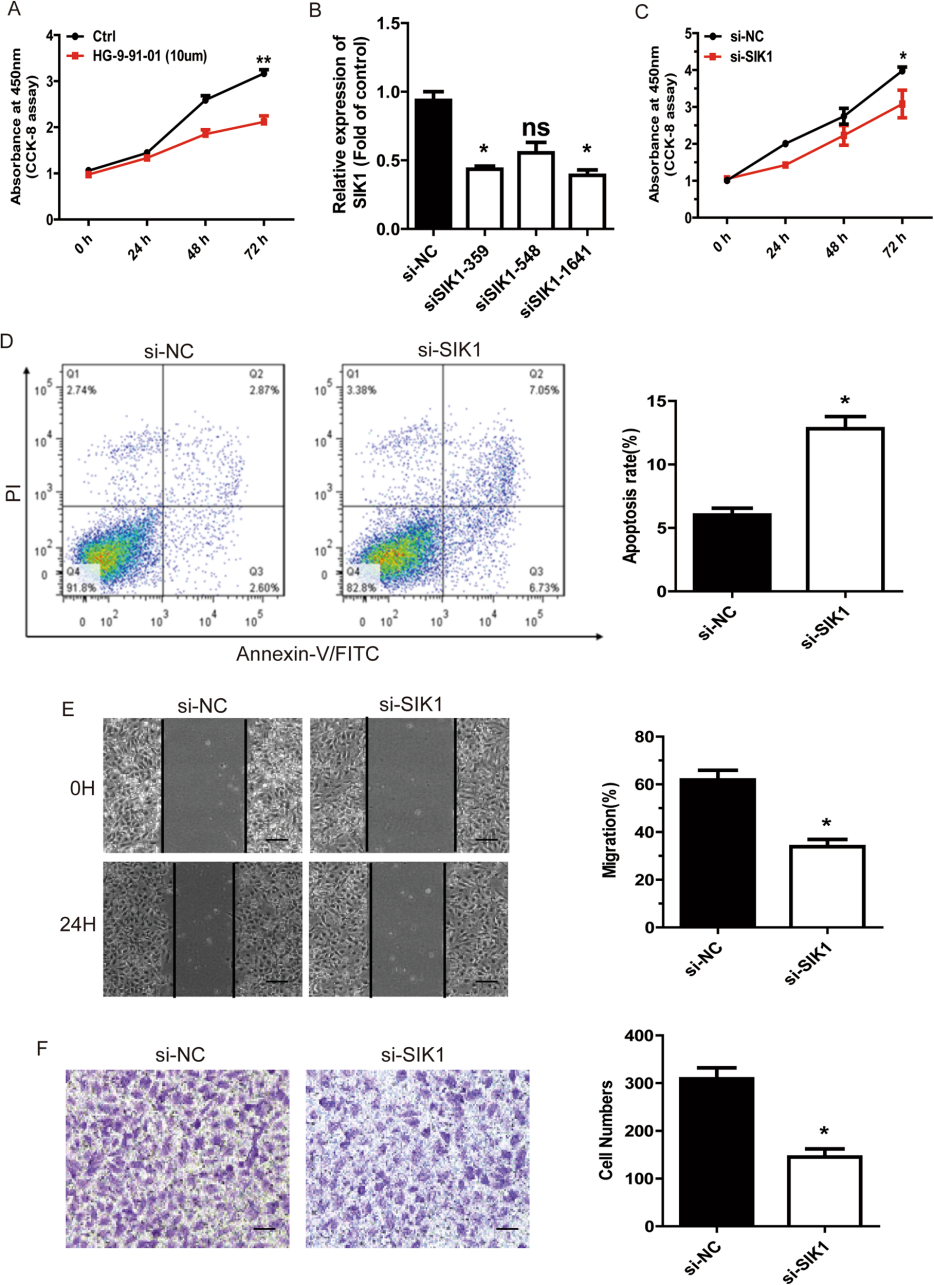
miR-130b-3p suppresses MB cell tumorigenesis through SIK1. **a** CCK-8 assay in MB cells treated with SIK1 inhibitor. **b** The efficiency of si-SIK1 knockdown was detected by RT-qPCR. **c** The proliferative ability of MB cells transfected with si-SIK1 or NC was tested through CCK-8. **d** Cell apoptosis was detected through flow cytometry in MB cells treated with si-SIK1 or negative control. **e** Cell migration was detected through wound-healing assay in MB cells transfected with si-SIK1 (original magnifications ×200). **f** Cell invasion analysis was performed through transwell invasion assay in MB cells transfected with si-SIK1 (original magnifications ×200). Data represent means ± SEMs from three independent experiments. *p < 0.05, **p < 0.01.

To test whether SIK1 was responsible for the reduced cell viability after miR-130b-3p mimic treatment, we measured cell proliferation following treatment using the SIK1 inhibitor HG-9-91-01. We found that HG-9-91-01 could significantly reduce cell proliferation, and this suggested that SIK1 itself could promote cell viability (Fig. 5a). Then, we knocked down SIK1 gene expression in Daoy cells using specific siRNAs. siSIK1-1641 was selected for the following experiments because of its significant knockdown efficiency (Fig. 5b). The suppression of SIK1 markedly decreased cell viability (Fig. 5c; Supplementary Fig. S4b) and increased annexin V-positive cell populations in Daoy cells (Fig. 5d). Therefore, the results from both the cell viability assay and the apoptosis assay upon si-SIK1 treatment were generally consistent with the results of the miR-130b-3p mimic treatment.

We also tested if SIK1 would affect cell motility. Daoy cells were transfected with si-SIK1 or NC. Twenty-four-hour wound-healing assay revealed that si-SIK1 effectively suppressed the migration capacity of Daoy cells compared with NC treatment (Fig. 5e). The transwell assay showed that si-SIK1 could reduce Daoy cell invasion (Fig. 5f). In conclusion, these results indicate that SIK1 promoted cell motility in MB cells in vitro and miR-130b-3p could reduce cell viability, promote cell apoptosis and inhibit cell motility in an MB cell line by targeting SIK1.

### miR-130b-3p promotes the activity of the p53 signaling pathways through downregulation of SIK1

**Fig. 6:**
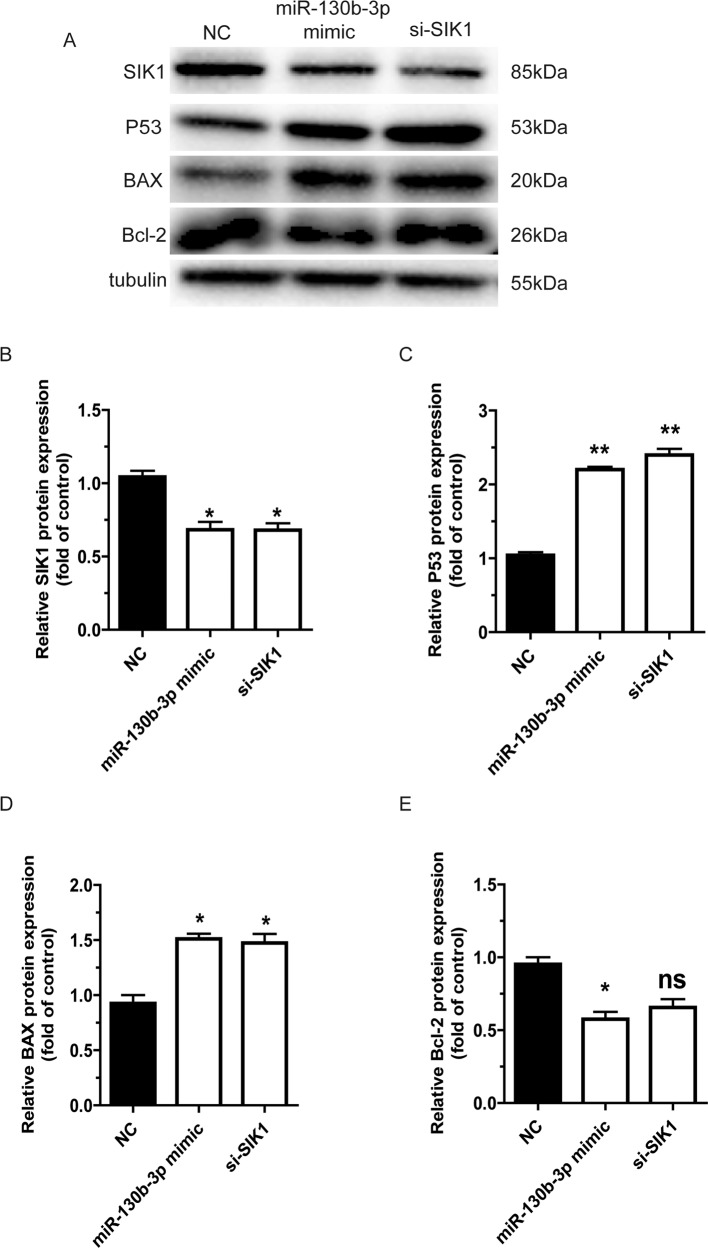
miR-130b-3p promotes the activity of the p53 signaling pathways through downregulation of SIK1. **a** The protein levels of SIK1, p53, BAX and Bcl-2 in Daoy cells were measured through western blotting. **b**–**e** SIK1, p53, BAX and Bcl-2 protein expression were determined by western blotting, respectively, in Daoy cells. Data represent means ± SEMs from three independent experiments. *p < 0.05, **p < 0.01.

We further investigated the mechanism through which miR-130b-3p regulates cell apoptosis in MB. The p53 pathways were reported to be involved in the LKB1 pathway and are important in cell apoptosis. We speculated that miR-130b-3p-SIK1 promoted apoptosis via the p53 signaling pathways. In Daoy cells, p53 and BAX were upregulated by miR-130b-3p overexpression and SIK1 knockdown, while Bcl-2 was downregulated (Fig. 6a–e). Therefore, these findings suggest that miR-130b-3p promotes the activity of the p53 signaling pathways through downregulation of SIK1 to regulate MB cell apoptosis.

### miR-130b-3p suppresses and SIK1 promotes MB tumorigenesis in vivo

**Fig. 7:**
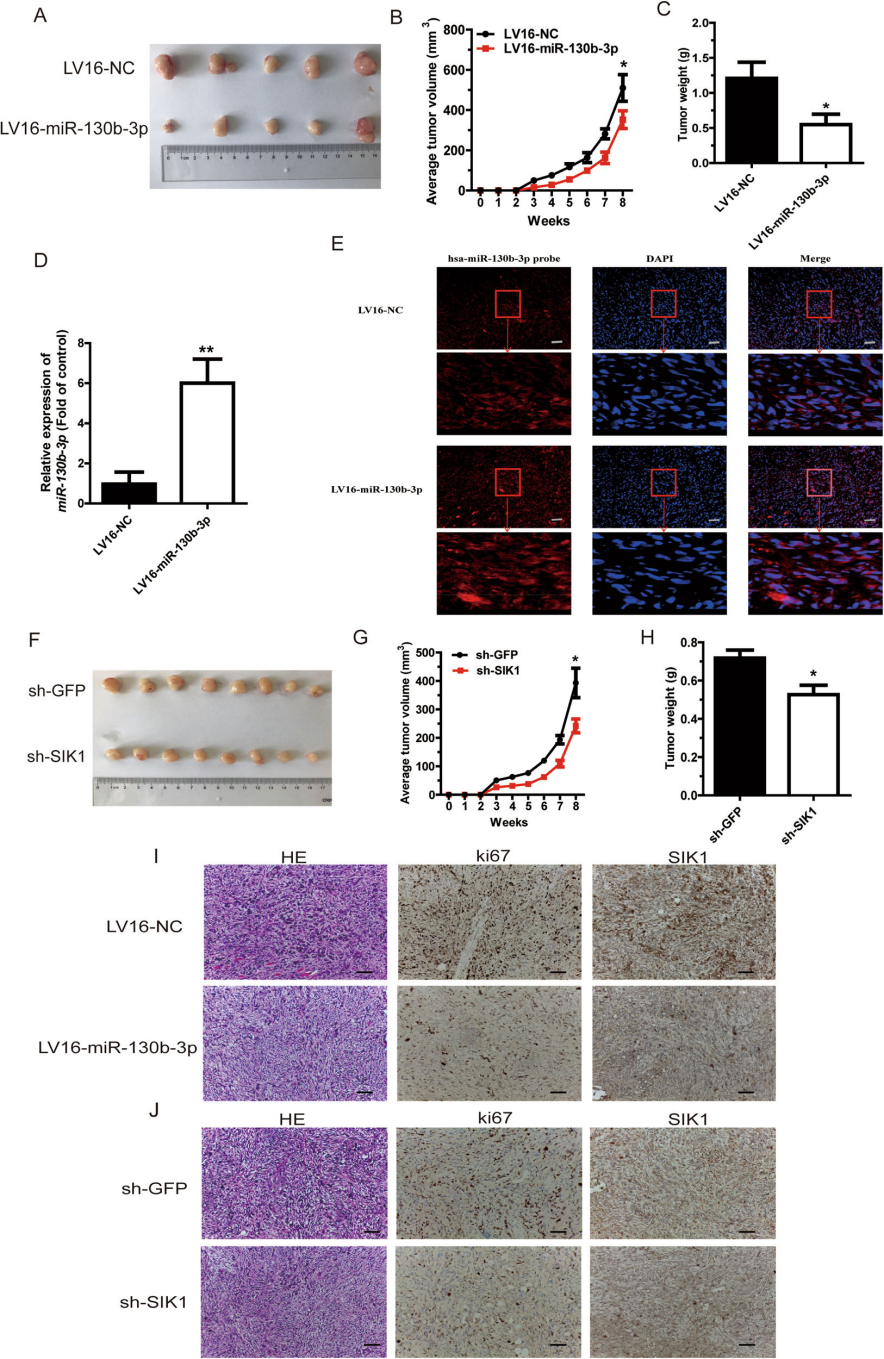
miR-130b-3p suppresses and SIK1 promotes MB tumorigenesis in vivo. **a** Nude mice were injected with subcutaneous xenografts of Daoy cells overexpressing miR-130b-3p or NC and were euthanized after 8 weeks (five mice per group). **b** Tumor volume was recorded weekly after an injection of Daoy cells transfected with LV16-miR-130b-3p and LV16-NC. **c** Tumor weight was measured 8 weeks post injection with Daoy cells transfected with LV16-miR-130b-3p and LV16-NC. **d**, **e** miR-130b-3p expression was measured in mice tumors by RT-qPCR and FISH. **f** Mouse tumors were imaged 8 weeks after injection with Daoy cells, and demonstrated decreasing SIK1 expression (eight mice per group). **g** Tumor volume was recorded weekly after injection with Daoy cells transfected with sh-SIK1 and sh-GFP. **h** Tumor weight was measured 8 weeks post injection with Daoy cells transfected with sh-SIK1 or sh-GFP. **i**, **j** Hematoxylin and eosin (HE) staining (original magnifications ×200) of tumors and SIK1 and Ki67 expression in the tumor tissue samples, as determined through IHC. Data represent means ± SEMs from three independent experiments. *p < 0.05, **p < 0.01.

To examine the role of miR-130b-3p and SIK1 in MB tumorigenesis in vivo, we constructed a xenograft animal model by implanting Daoy cells with stable overexpression of miR-130b-3p or knockdown of SIK1 in 6-week-old nude mice. The mice were euthanized after 8 weeks. Tumor volume was significantly smaller in the miR-130b-3p overexpression group (LV16-miR-130b-3p) than in the NC group (LV16-miR-NC). Moreover, tumor weight was also lower in mice inoculated with Daoy-LV16-miR-130b-3p cells compared with those in the control group at week 8 (Fig. 7a–c). Furthermore, miR-130b-3p expression was significantly upregulated by RT-qPCR and FISH (Fig. 7d, e). Similarly, in mice inoculated with sh-SIK1 cells, the average tumor volume was substantially smaller than in sh-GFP mice at week 8, and the tumor weight was also lower (Fig. 7f–h). Xenografts were also subjected to IHC assays. The results showed that SIK1 expression decreased in the LV16-miR-130b-3p-treated group, similar to the sh-SIK1-treated group (Fig. 7i, j). Ki-67 staining of the xenograft tumors was also performed, and the results further confirmed the inhibitory effect of miR-130b-3p and the promotive effect of SIK1 on MB tumorigenesis (Fig. 7i, j).

## Discussion

**Fig. 8:**
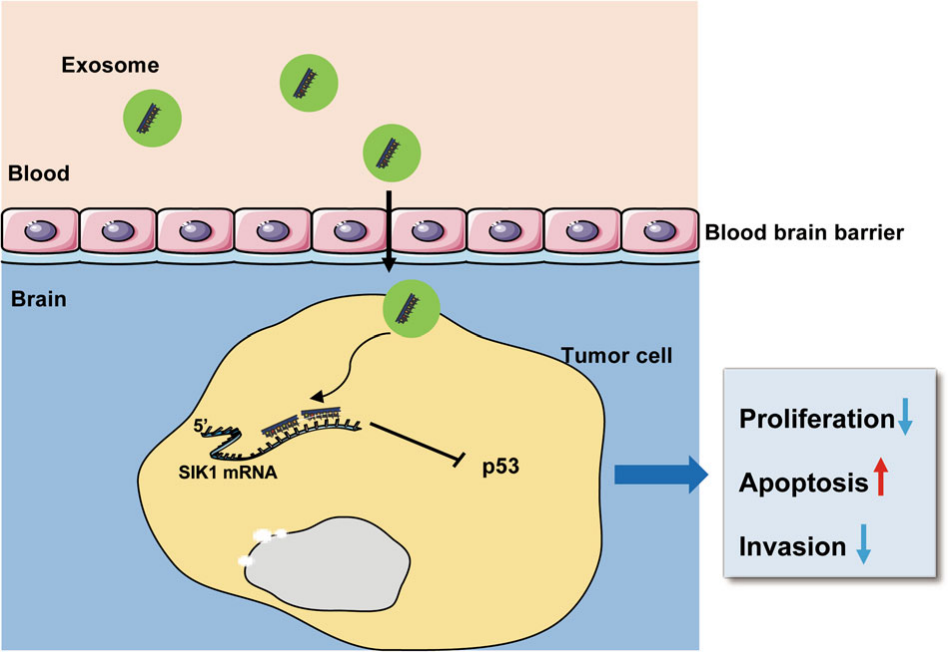
miR-130b-3p promotes the activity of p53 signaling pathways through downregulation of SIK1. Schematic model of the role of miR-130b-3p in regulating MB tumorigenesis.

Exosomes are an important carrier in cell communication^10^. Although exosomes have been studied for several years, the biological roles of exosomal miRNAs are just beginning to be investigated. miRNAs also serve as important intercellular communication tools, as they are transferred between cells via exosomes and influence the phenotypes of their recipient cells^26–28^. In the current study, we previously analyzed the miRNA expression profiles of exosomes derived from MB patient plasma, and found that there was a higher level of miR-130b-3p in exosomes derived from MB patient plasma than from exosomes derived from healthy control plasma. Exosomal miR-130b-3p from MB patient plasma could be transferred to the MB cell line and played a tumor suppressor role. miR-130b-3p suppressed MB tumorigenesis by targeting a novel target, SIK1, through the p53 signaling pathways (Fig. 8). Our research provides new insight into the molecular mechanism of MB and may offer a new therapeutic strategy for MB.

In this research, the expression of miR-130b-3p was higher in circulating exosomes while lower in tumor tissues. This phenomenon was also reported in several other cancers, like lung adenocarcinoma^29,30^, esophagus adenocarcinoma^31^, intestinal-type sinonasal adenocarcinoma^32^, and ovarian cancer^33^. The plausible explanation is exosomes derived from normal cells can act as a carrier for delivery of antitumor factors. But the detailed mechanism is needed for further analysis. To study the origin of the exosomes containing miR-130b-3p, we isolated exosomes from MB patient plasma and THP-1 culture supernatant that was transfected with miR-130b-3p mimic, and we found there was higher expression of miR-130b-3p in the isolated exosomes. So we suspected that monocyte in the PBMCs was one source of the exosomes containing miR-130b-3p. We also found that exosomes from THP-1 could also transfer into MB cells, and the expression of miR-130b-3p was higher in cocultured with exosomes derived from THP-1. While this phenomenon disappeared after treatment with GW4869. It was known that monocyte could migrate into tissues and transformed into macrophages^34^. Macrophages were a kind of innate immune cells that played an important role in maintaining homeostasis and host defense^35^, especially in the progression from early cancer to metastatic tumor^36,37^. Yin et al.^38^ reported that macrophage-derived exosomes played a vital role in the development of tumor. So, the plausible explanation is exosomes derived from mono-macrophage cells can act as a carrier for delivery of antitumor factors. As for how the exosomal miR-130b-3p derived from mono-macrophages, further research is needed.

miR-130b-3p has been shown to have different effects in different cancer types, and functions as an oncogene in thyroid adenomas^23^ and epithelial ovarian cancer^39^. miR-130b-3p also acts as a tumor suppressor in breast carcinoma^22^ and ovarian cancer^40^. However, to date, there has been no research concerning the function of miR-130b-3p in MB. In the current study, we have demonstrated that miR-130b-3p played an important suppressive role in the progression of MB. In this study, we found that exosomal miR-130b-3p in MB patient plasma was upregulated than in healthy, but we did not further distinguish whether the expression levels of miR-130b-3p in each molecular subgroup according to current WHO classification was different due to the limitation of the number of MB patients. In the future, a large population of MB patients is needed to evaluate whether exosomal miR-130b-3p can serve as a biomarker for MB diagnosis and prognosis.

In this study, we confirmed that SIK1 was an unreported direct target of miR-130b-3p using a dual luciferase reporter assay. SIK1, a serine/threonine kinase, is a member of the AMPK family, which plays vital roles in the regulation of cell growth^41^. Recent evidence has shown that SIK1 performs an important role in human cancers, such as non-small-cell lung cancer^42^, epithelial ovarian cancer^43^ or hepatocellular carcinoma^41,44^. Previous research has shown that SIK1 is a tumor suppressor in many cancers^45,46^. Interestingly, in the current study, we identified SIK1 as a tumor-promoting protein, as it can induce tumor cell proliferation, invasion and metastasis in vivo/vitro. We further found that the overexpression of miR-130b-3p could upregulate the protein expression of p53 and BAX, while downregulating Bcl-2. Therefore, our results indicate that miR-130b-3p may function as a tumor suppressor by inhibiting SIK1 protein expression and subsequent downstream p53 activation in MB cell lines, which might provide a new therapeutic strategy for MB.

In conclusion, the current study demonstrates that the intracellular miR-130b-3p affects the proliferation, migration and invasiveness of MB, which is mediated through targeting SIK1, and exosomal miR-130b-3p play a tumor suppressor role in MB tumorigenesis. However, further research is needed to understand the more profound molecular mechanism of exosomal miR-130b-3p origin and function in MB tumorigenesis.

## Supplementary information

supplementary-table

Fig S1

Fig S2

Fig S3

Fig S4
