# Supplementary material for: Exosomal miR-130b-3p targets SIK1 to inhibit medulloblastoma tumorigenesis
Source: Cell Death Dis. 2020 Jun 1;11(6):408. doi: 10.1038/s41419-020-2621-y (PMC7264172; doi:10.1038/s41419-020-2621-y)
Supplement: Supplementary file 1 — supplementary-table [file 41419_2020_2621_MOESM1_ESM.docx]

**Supplementary figure legends**

**Fig S1. The heatmap of differential expressed miRNAs in exosomes derived from MB patients and healthy controls plasma.** The color scale shows the relative expression of miRNAs across different samples (fold-change >1.5, n=3).

**Fig S2. The expression of miR-130b-3p and SIK1 in tissues of MB.** RT-qPCR analysis was performed to evaluate the expression of miR-130b-3p and SIK1 mRNA in MB tissues and adjacent non-tumor tissues. Data are presented as mean ± SEM. **p* < 0.05.

**Fig S3. HMO6 derived exosomal miR-130b-3p can be transferred to MB cells.**

**A,** Exosomes were isolated from culture supernatant from HMO6 cells, dyed with PKH67 (green) and co-cultured with Daoy cells for 12h, then dyed with Hoechst33258 (blue) and viewed with confocal microscopy (original magnifications×200). **B,** The expression of miR-130b-3p in Daoy cells co-cultured with the exosomes collected from HMO6 culture supernatant, detected by RT-qPCR. **C,** Immunohistochemistry (original magnifications×200) analysis of CD68 in MB patients. Data represent means ± SEMs from three independent experiments. **p* < 0.05.

**Fig S4. The colony formation of MB cells. A,** The proliferative ability of MB cells transfected with miR-130b-3p mimic or negative control was tested through cell colony formation assays. **B,** The proliferative ability of MB cells transfected with siSIK1 or negative control was tested through cell colony formation assays. Data represent means ± SEMs from three independent experiments. **p* < 0.05, ***p* < 0.01.

**Supplementary tables**

**Table S1** Detail information of medulloblastoma patients enrolled in the study

| No. | Sex | Age(m) | Histology | Subgroup | Metastasis(Y/N) |
| --- | --- | --- | --- | --- | --- |
| 1 | M | 72 | Desmoplastic/nodular | SHH | N |
| 2 | M | 29 | Classic | Group 4 | Y |
| 3 | M | 27 | Desmoplastic | SHH | N |
| 4 | M | 108 | Classic | Group 4 | N |
| 5 | M | 96 | Classic | WNT | N |
| 6 | M | 39 | Classic | Group 4 | N |
| 7 | F | 120 | Classic | Group 4 | N |
| 8 | M | 44 | Classic | WNT | N |
| 9 | M | 96 | Classic | Group 4 | Y |
| 10 | F | 108 | Classic | Group 4 | N |
| 11 | M | 43 | Classic | WNT | Y |
| 12 | M | 25 | Classic | Group 4 | Y |
| 13 | F | 96 | Classic | Group 3 | N |
| 14 | M | 5 | Desmoplastic/Classic | WNT | N |
| 15 | M | 22 | Classic | Group 3 | Y |
| 16 | M | 120 | Classic | SHH | N |
| 17 | F | 120 | Desmoplatic | SHH | N |
| 18 | F | 19 | Desmoplatic | Group 4 | Y |
| 19 | M | 32 | Classic | Group 3 | Y |
| 20 | M | 48 | Extensive nodularity | Group 4 | N |
| 21 | M | 14 | Classic | WNT | N |
| 22 | M | 56 | Classic | Group 4 | N |
| 23 | F | 132 | Classic | Group 3 | N |
| 24 | F | 12 | Extensive nodularity | SHH | N |
| 25 | F | 156 | Classic | WNT | Y |

**Table S2** Sequences of NC siRNA, siRNA and mimic

|  | **Sense sequence (5’-3’)** |
| --- | --- |
| NC | UUCUCCGAACGUGUCACGUTT |
| miR-130b-3p mimic | CAGUGCAAUGAUGAAAGGGCAU |
| siSIK1-1641 | GGAACCAGCUCUGACAGUUTT |

**Table S3** Primer sequences used for amplification

|  | **Sense sequence (5’-3’)** |
| --- | --- |
| hsa-miR-130b-3p | CAGUGCAAUGAUGAAAGGGCAU |
| U6 | GUGCUCGCUUCGGCAGCACAUAUACUAAAAUUGGAACGAUACAGAGAAGAUUAGCAUGGCCCCUGCGCAAGGAUGACACGCAAAUUCGUGAAGCGUUCCAUAUUUU |
| SIK1 | Forward: GCTCAAGGAGTATCGGAATGCC  Reverse: GTGGAAAGACCTTCCTGAGGCA |
| SIK3 | Forward: CTCAGCCATCTCCACCTCTTCA  Reverse: GGCTGCCTGAAGAGATGGTTGT |
| ESR1 | Forward: GCTTACTGACCAACCTGGCAGA  Reverse: GGATCTCTAGCCAGGCACATTC |
| SMAD4 | Forward: CTACCAGCACTGCCAACTTTCC  Reverse: CCTGATGCTATCTGCAACAGTCC |
| MAP2 | Forward: AGGCTGTAGCAGTCCTGAAAGG  Reverse: CTTCCTCCACTGTGACAGTCTG |
| TSC1 | Forward: CTGGACAGACTGATACAGCAGG  Reverse: TGCGGATCTCATCTGAAGGAGG |
| β-actin | Forward: CACCATTGGCAATGAGCGGTTC  Reverse: AGGTCTTTGCGGATGTCCACGT |
