# Supplementary figures and images for: Exosomal miR-130b-3p targets SIK1 to inhibit medulloblastoma tumorigenesis
Source: Cell Death Dis. 2020 Jun 1;11(6):408. doi: 10.1038/s41419-020-2621-y (PMC7264172; doi:10.1038/s41419-020-2621-y)

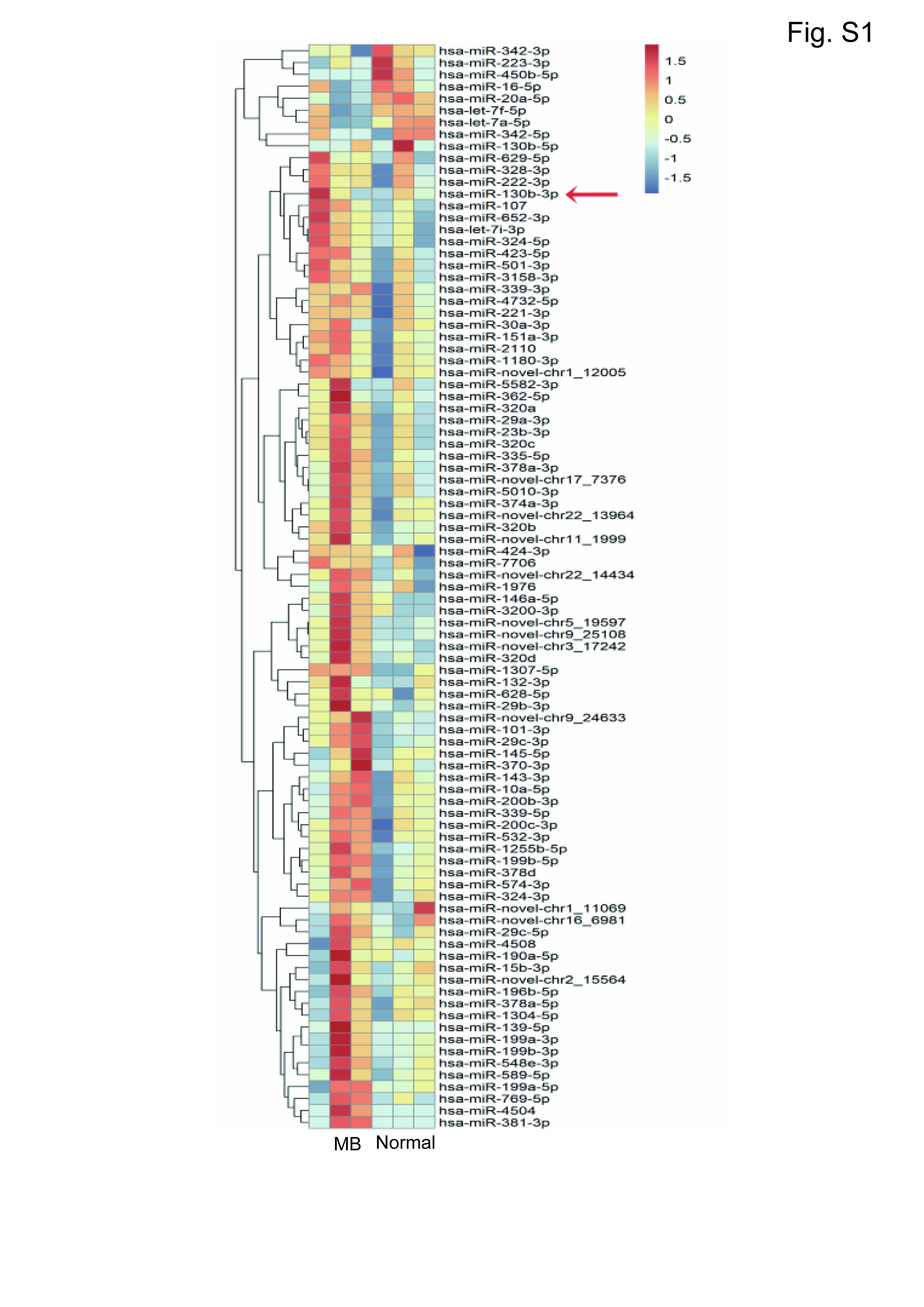

Supplement: Supplementary file 2 — Fig S1 [file 41419_2020_2621_MOESM2_ESM.tif]

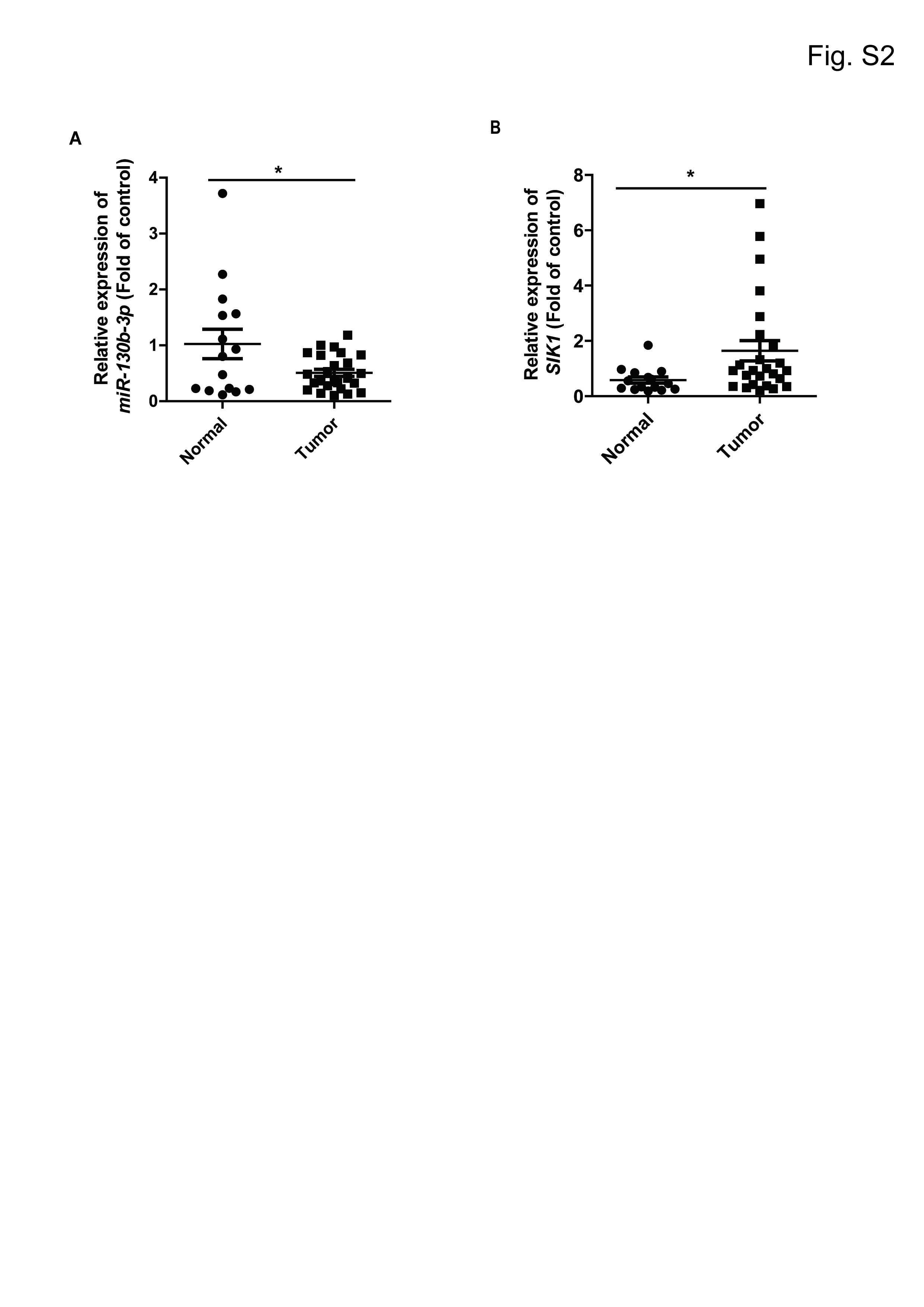

Supplement: Supplementary file 3 — Fig S2 [file 41419_2020_2621_MOESM3_ESM.tif]

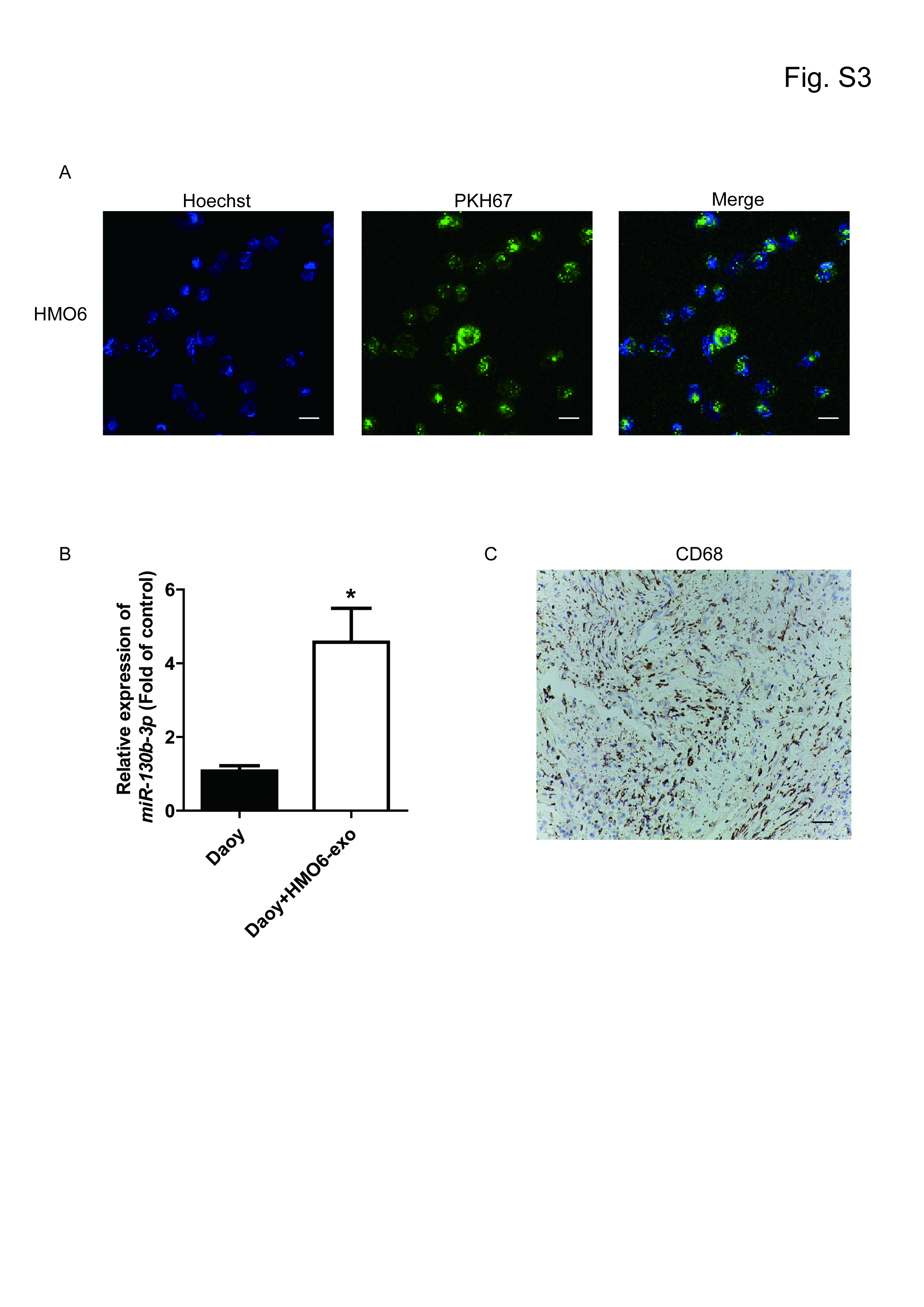

Supplement: Supplementary file 4 — Fig S3 [file 41419_2020_2621_MOESM4_ESM.tif]

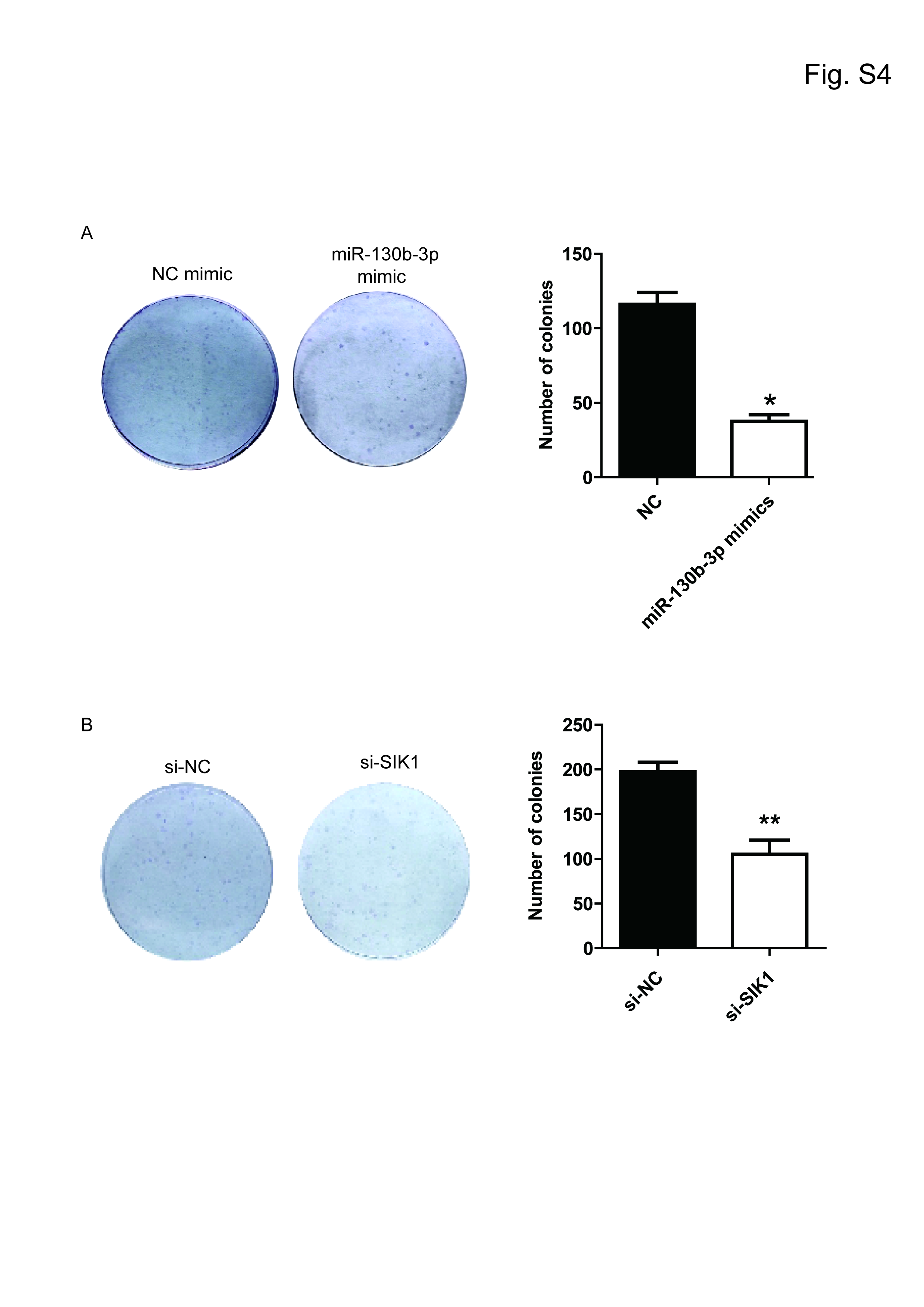

Supplement: Supplementary file 5 — Fig S4 [file 41419_2020_2621_MOESM5_ESM.tif]
